# On a reverse Mulholland’s inequality in the whole plane

**DOI:** 10.1186/s13660-018-1634-x

**Published:** 2018-02-14

**Authors:** Aizhen Wang, Bicheng Yang

**Affiliations:** Department of Mathematics, Guangdong University of Education, Guangzhou, P.R. China

**Keywords:** 26D15, 47A07, Mulholland’s inequality, Parameter, Weight coefficient, Equivalent form, Reverse

## Abstract

By introducing multi-parameters, applying the weight coefficients and Hermite–Hadamard’s inequality, we give a reverse of the extended Mulholland inequality in the whole plane with the best possible constant factor. The equivalent forms and a few particular cases are also considered.

## Introduction

Assuming that $p>1$, $\frac{1}{p}+\frac{1}{q}=1$, $a_{n},b_{n}\geq0$, $0<\sum_{n=1}^{\infty}a_{n}^{p}<\infty$, and $0<\sum_{n=1}^{\infty }b_{n}^{q}<\infty$, we have the following Hardy–Hilbert inequality (cf. [1]):

1 $$ \sum_{n=1}^{\infty}\sum _{m=1}^{\infty}\frac{a_{m}b_{n}}{m+n}< \frac{\pi}{\sin(\pi/p)} \Biggl( \sum_{n=1}^{\infty}a_{n}^{p} \Biggr) ^{1/p} \Biggl( \sum_{n=1}^{\infty}b_{n}^{q} \Biggr) ^{1/q}, $$

where the constant factor $\frac{\pi}{\sin(\pi/p)}$ is the best possible. We still have the following Mulholland inequality with the same best possible constant factor $\frac{\pi}{\sin(\pi/p)}$ (cf. Theorem 343 of [1], replacing $\frac{a_{m}}{m}$, $\frac{b_{n}}{n}$ by $a_{m}$, $b_{n}$):

2 $$ \sum_{n=2}^{\infty}\sum _{m=2}^{\infty}\frac{a_{m}b_{n}}{\ln mn}< \frac{\pi }{\sin(\pi/p)} \Biggl( \sum_{n=2}^{\infty}\frac{a_{m}^{p}}{m^{1-p}} \Biggr) ^{1/p} \Biggl( \sum_{n=2}^{\infty} \frac{b_{n}^{q}}{n^{1-q}} \Biggr) ^{1/q}. $$

Inequalities (1)–(2) are important in the analysis and its applications (cf. [1, 2]).

In 2007, Yang [3] first gave a Hilbert-type integral inequality in the whole plane as follows:

3 $$\begin{aligned} & \int_{-\infty}^{\infty} \int_{-\infty}^{\infty}\frac{f(x)g(y)}{(1+e^{x+y})^{\lambda}}\,dx\,dy \\ &\quad < B \biggl(\frac{\lambda}{2},\frac{\lambda}{2} \biggr) \biggl( \int_{-\infty}^{\infty }e^{-\lambda x}f^{2}(x)\,dx \int_{-\infty}^{\infty}e^{-\lambda y}f^{2}(y)\,dy \biggr)^{\frac{1}{2}}, \end{aligned}$$

where the constant factor $B(\frac{\lambda}{2},\frac{\lambda}{2})$ ($\lambda>0$) is the best possible. Some new results on inequalities (1)–(3) were provided by [4–24]. In 2016, Yang and Chen [25] gave a more accurate extension of (1) in the whole plane as follows:

4 $$\begin{aligned} &\sum_{ \vert n \vert =1}^{\infty}\sum _{ \vert m \vert =1}^{\infty}\frac{a_{m}b_{n}}{ ( \vert m-\xi \vert + \vert n-\eta \vert ) ^{\lambda}} \\ &\quad < 2B ( \lambda _{1},\lambda_{2} ) \Biggl[ \sum _{ \vert m \vert =1}^{\infty} \vert m-\xi \vert ^{p(1-\lambda_{1})-1}a_{m}^{p} \Biggr] ^{\frac{1}{p}} \Biggl[ \sum_{ \vert n \vert =1}^{\infty} \vert n-\eta \vert ^{q(1-\lambda_{2})-1}b_{n}^{q} \Biggr] ^{\frac{1}{q}}, \end{aligned}$$

where the constant factor $2B ( \lambda_{1},\lambda_{2} ) $ ($0<\lambda_{1},\lambda_{2}\leq1$, $\lambda_{1}+\lambda_{2}=\lambda $, $\xi , \eta\in{[} 0,\frac{1}{2}]$) is the best possible. Another result was provided by Xin et al. [26].

In this paper, by introducing multi-parameters, applying the weight coefficients and Hermite–Hadamard’s inequality, we give a reverse of the extension of Mulholland’s inequality (2) in the whole plane with the best possible constant factor similar to the reverse of (4). The equivalent forms and a few particular cases are also considered.

## Some lemmas

In the following, we agree that $0< p<1$, $\frac{1}{p}+\frac{1}{q}=1$, $\lambda_{1},\lambda_{2}>0$, $\lambda_{1}+\lambda_{2}=\lambda\leq 1$, $\alpha,\beta\in{[} \arccos\frac{1}{3},\frac{\pi}{2}]$, $\xi ,\eta \in{[} 0,\frac{1}{2}]$, and

5 $$ H_{\gamma}(\lambda_{1}):=\frac{2\pi\csc^{2}\gamma}{\lambda\sin (\frac{\pi\lambda_{1}}{\lambda})}\quad (\gamma= \alpha,\beta). $$

### Remark 1

In view of the conditions that $\alpha,\beta\in{[} \arccos\frac{1}{3},\frac{\pi}{2}]$, $\xi,\eta\in{[} 0,\frac{1}{2}]$, it follows that $(\frac{3}{2}\pm\eta)(1\mp\cos\beta)\geq1$ and $(\frac{3}{2}\pm\xi)(1\mp\cos\alpha)\geq1$.

We set the following functions:

$$ A_{\alpha}(\xi,x)= \vert x-\xi \vert +(x-\xi)\cos\alpha, $$

$B_{\beta}(\eta,y)= \vert y-\eta \vert +(y-\eta)\cos\beta$, and

6 $$ H(x,y):=\frac{1}{\ln^{\lambda}A_{\alpha}(\xi,x)+\ln^{\lambda }B_{\beta }(\eta,y)}\quad \biggl( \vert x \vert , \vert y \vert > \frac{3}{2} \biggr). $$

### Definition 1

Define two weight coefficients as follows:

7 $$\begin{aligned} &\omega(\lambda_{2},m):=\sum_{ \vert n \vert =2}^{\infty} \frac{H(m,n)}{B_{\beta}(\eta,n)}\frac{\ln^{\lambda_{1}}A_{\alpha}(\xi ,m)}{\ln^{1-\lambda_{2}}B_{\beta}(\eta,n)}, \quad \vert m \vert \in \mathbf{N} \backslash \{1\}, \end{aligned}$$

8 $$\begin{aligned} &\varpi(\lambda_{1},n):=\sum_{ \vert m \vert =2}^{\infty} \frac{H(m,n)}{A_{\alpha}(\xi,m)}\frac{\ln^{\lambda_{2}}B_{\beta}(\eta ,n)}{\ln^{1-\lambda_{1}}A_{\alpha}(\xi,m)}, \quad \vert n \vert \in \mathbf{N} \backslash \{1\}, \end{aligned}$$

where $\sum_{ \vert j \vert =2}^{\infty}\cdots =\sum_{j=-2}^{-\infty}\cdots+\sum_{j=2}^{\infty}\cdots$ ($j=m,n$).

### Lemma 1

(cf. [26])

*Suppose that* $g(t)$ (>0) *is strictly decreasing in* $(1,\infty)$, *satisfying* $\int_{1}^{\infty}g(t)\,dt\in \mathbf{R}_{+}$. *We have*

9 $$ \int_{2}^{\infty}g(t)\,dt< \sum _{n=2}^{\infty}g(n)< \int_{1}^{\infty}g(t)\,dt. $$

*If* $(-1)^{i}g(t)>0$ ($i=0,1,2$; $t\in(\frac{3}{2},\infty)$), $\int_{\frac {3}{2}}^{\infty}g(t)\,dt\in\mathbf{R}_{+}$, *then we have the following Hermite–Hadamard inequality* (*cf*. [27]):

10 $$ \sum_{n=2}^{\infty}g(n)< \int_{\frac{3}{2}}^{\infty}g(t)\,dt. $$

### Lemma 2

*For* $0<\lambda\leq1$, $0<\lambda_{2}<1$, *the following inequalities are valid*:

11 $$ H_{\beta}(\lambda_{1}) \bigl(1-\theta(\lambda _{2},m) \bigr)< \omega(\lambda_{2},m)< H_{\beta}( \lambda_{1}),\quad \vert m \vert \in\mathbf{N}\backslash \{1\}, $$

*where*

12 $$\begin{aligned} \theta(\lambda_{2},m) :=&\frac{\lambda}{\pi}\sin \biggl( \frac{\pi\lambda_{1}}{\lambda} \biggr) \int_{0}^{\frac{\ln[(2+\eta)(1+\cos\beta)]}{\ln A_{\alpha }(\xi,m)}}\frac{u^{\lambda_{2}-1}}{1+u^{\lambda}}\,du \\ =&O \biggl( \frac{1}{\ln^{\lambda_{2}}A_{\alpha}(\xi,m)} \biggr) \in(0,1). \end{aligned}$$

### Proof

For $\vert m \vert \in\mathbf{N}\backslash\{ 1\}$, we set the following functions:

$$\begin{aligned} &H^{(1)}(m,y) :=\frac{1}{\ln^{\lambda}A_{\alpha}(\xi,m)+\ln ^{\lambda }[(y-\eta)(\cos\beta-1)]},\quad y< -\frac{3}{2}, \\ &H^{(2)}(m,y) :=\frac{1}{\ln^{\lambda}A_{\alpha}(\xi,m)+\ln ^{\lambda }[(y-\eta)(\cos\beta+1)]},\quad y>\frac{3}{2}, \end{aligned}$$

wherefrom

$$ H^{(1)}(m,-y)=\frac{1}{\ln^{\lambda}A_{\alpha}(\xi,m)+\ln ^{\lambda }[(y+\eta)(1-\cos\beta)]},\quad y>\frac{3}{2}. $$

We find

13 $$\begin{aligned} \omega(\lambda_{2},m) =&\sum_{n=-2}^{-\infty} \frac{H^{(1)}(m,n)\ln ^{\lambda_{1}}A_{\alpha}(\xi,m)}{(n-\eta)(\cos\beta-1)\ln ^{1-\lambda _{2}} [ (n-\eta)(\cos\beta-1) ] } \\ &{}+\sum_{n=2}^{\infty}\frac{H^{(2)}(m,n)\ln^{\lambda_{1}}A_{\alpha }(\xi ,m)}{(n-\eta)(1+\cos\beta)\ln^{1-\lambda_{2}} [ (n-\eta)(1+\cos \beta) ] } \\ =&\frac{\ln^{\lambda_{1}}A_{\alpha}(\xi,m)}{1-\cos\beta}\sum_{n=2}^{\infty} \frac{H^{(1)}(m,-n)}{(n+\eta)\ln^{1-\lambda _{2}}[(n+\eta)(1-\cos\beta)]} \\ &{}+\frac{\ln^{\lambda_{1}}A_{\alpha}(\xi,m)}{1+\cos\beta}\sum_{n=2}^{\infty} \frac{H^{(2)}(m,n)}{(n-\eta)\ln^{1-\lambda _{2}}[(n-\eta)(1+\cos\beta)]}. \end{aligned}$$

In virtue of $0<\lambda\leq1$, $0<\lambda_{2}<1$, we find that for $y>\frac{3}{2}$,

$$\begin{aligned} &\frac{d}{dy}\frac{H^{(i)}(m,(-1)^{i}y)}{(y-(-1)^{i}\eta)\ln ^{1-\lambda _{2}}[(y-(-1)^{i}\eta)(1+(-1)^{i}\cos\beta)]} < 0, \\ &\frac{d^{2}}{dy^{2}}\frac{H^{(i)}(m,(-1)^{i}y)}{(y-(-1)^{i}\eta)\ln ^{1-\lambda_{2}}[(y-(-1)^{i}\eta)(1+(-1)^{i}\cos\beta)]} >0\quad (i=1.2), \end{aligned}$$

it follows that $\frac{H^{(i)}(m,(-1)^{i}y)}{(y-(-1)^{i}\eta)\ln ^{1-\lambda_{2}}[(y-(-1)^{i}\eta)(1+(-1)^{i}\cos\beta)]}$ ($i=1.2$) are strictly decreasing and strictly convex in $(\frac{3}{2},\infty)$. By (10) and (13) we find

$$\begin{aligned} \omega(\lambda_{2},m) < &\frac{\ln^{\lambda_{1}}A_{\alpha}(\xi ,m)}{1-\cos\beta} \int_{3/2}^{\infty}\frac{H^{(1)}(m,-y)\,dy}{(y+\eta)\ln ^{1-\lambda_{2}}[(y+\eta)(1-\cos\beta)]} \\ &{}+\frac{\ln^{\lambda_{1}}A_{\alpha}(\xi,m)}{1+\cos\beta}\int_{3/2}^{\infty}\frac{H^{(2)}(m,y)\,dy}{(y-\eta)\ln^{1-\lambda _{2}}[(y-\eta)(1+\cos\beta)]}. \end{aligned}$$

Setting $u=\frac{\ln[(y+\eta)(1-\cos\beta)]}{\ln A_{\alpha}(\xi ,m)}$ ($u=\frac{\ln[(y-\eta)(1+\cos\beta)]}{\ln A_{\alpha}(\xi,m)}$) in the above first (second) integral, in view of Remark 1, by simplifications, we obtain

$$\begin{aligned} \omega(\lambda_{2},m) < & \biggl( \frac{1}{1-\cos\beta}+ \frac{1}{1+\cos \beta} \biggr) \int_{0}^{\infty}\frac{u^{\lambda_{2}-1}}{1+u^{\lambda}}\,du \\ =&\frac{2\csc^{2}\beta}{\lambda} \int_{0}^{\infty}\frac{v^{(\lambda _{2}/\lambda)-1}\,dv}{1+v}= \frac{2\pi\csc^{2}\beta}{\lambda\sin(\frac{\pi\lambda_{1}}{\lambda})}=H_{\beta}(\lambda_{1}). \end{aligned}$$

By (9) and (13), in the same way, we still have

$$\begin{aligned} \omega(\lambda_{2},m) >&\frac{\ln^{\lambda_{1}}A_{\alpha}(\xi ,m)}{1-\cos\beta} \int_{2}^{\infty}\frac{H^{(1)}(m,-y)\,dy}{(y+\eta)\ln ^{1-\lambda_{2}}[(y+\eta)(1-\cos\beta)]} \\ &{}+\frac{\ln^{\lambda_{1}}A_{\alpha}(\xi,m)}{1+\cos\beta}\int_{2}^{\infty}\frac{H^{(2)}(m,y)\,dy}{(y-\eta)\ln^{1-\lambda _{2}}[(y-\eta)(1+\cos\beta)]} \\ \geq& \biggl( \frac{1}{1-\cos\beta}+\frac{1}{1+\cos\beta} \biggr) \int_{\frac{\ln[(2+\eta)(1+\cos\beta)]}{\ln A_{\alpha}(\xi ,m)}}^{\infty}\frac{u^{\lambda_{2}-1}}{1+u^{\lambda}}\,du \\ =&H_{\beta}(\lambda_{1})-2\csc^{2}\beta \int_{0}^{\frac{\ln[(2+\eta )(1+\cos\beta)]}{\ln A_{\alpha}(\xi,m)}}\frac{u^{\lambda_{2}-1}}{1+u^{\lambda}}\,du \\ =&H_{\beta}(\lambda_{1}) \bigl(1-\theta(\lambda _{2},m) \bigr)>0, \end{aligned}$$

where $\theta(\lambda_{2},m)$ is indicated by (12). It follows that $\theta(\lambda_{2},m)<1$ and

$$\begin{aligned} 0 < &\theta(\lambda_{2},m) \\ < &\frac{\lambda}{\pi}\sin \biggl( \frac{\pi\lambda _{1}}{\lambda} \biggr) \int_{0}^{\frac{\ln[(2+\eta)(1+\cos\beta)]}{\ln A_{\alpha}(\xi,m)}}u^{\lambda_{2}-1}\,du \\ =&\frac{\lambda}{\pi\lambda_{2}}\sin \biggl(\frac{\pi\lambda _{1}}{\lambda} \biggr) \biggl( \frac{\ln[(2+\eta)(1+\cos\beta)]}{\ln A_{\alpha}(\xi,m)} \biggr) ^{\lambda_{2}}. \end{aligned}$$

Hence, (11) and (12) are valid. □

In the same way, for $0<\lambda\leq1$, $0<\lambda_{1}<1$, we still have the following.

### Lemma 3

*The following inequalities are valid*:

14 $$ H_{\alpha}(\lambda_{1}) \bigl(1-\widetilde{\theta}(\lambda _{1},n) \bigr)< \varpi(\lambda_{1},n)< H_{\alpha}( \lambda_{1}), \quad \vert n \vert \in\mathbf{N}\backslash\{1\}, $$

*where*

15 $$\begin{aligned} \widetilde{\theta}(\lambda_{1},n) :=&\frac{\lambda}{\pi}\sin \biggl( \frac{\pi \lambda_{1}}{\lambda} \biggr) \int_{0}^{\frac{\ln[(2+\xi)(1+\cos\alpha)]}{\ln B_{\beta}(\eta,n)}}\frac{u^{\lambda_{1}-1}}{1+u^{\lambda}}\,du \\ =&O \biggl( \frac{1}{\ln^{\lambda_{1}}B_{\beta}(\eta,n)} \biggr) \in(0,1). \end{aligned}$$

### Lemma 4

*If* $(\zeta,\gamma)=(\xi,\alpha)$ (*or* $(\eta ,\beta)$), $\rho>0$, *then we have*

16 $$\begin{aligned} h_{\rho}(\zeta,\gamma) :=&\sum_{ \vert n \vert =2}^{\infty} \frac{\ln^{-1-\rho} [ \vert n-\zeta \vert +(n-\zeta)\cos\gamma ] }{ [ \vert n-\zeta \vert +(n-\zeta)\cos\gamma ] } \\ =&\frac{1}{\rho} \bigl(2\csc^{2}\gamma+o(1) \bigr)\quad \bigl( \rho\rightarrow0^{+} \bigr) . \end{aligned}$$

### Proof

Still by (10) we find

$$\begin{aligned} h_{\rho}(\zeta,\gamma) =&\sum_{n=-2}^{-\infty} \frac{\ln^{-1-\rho }[(n-\zeta)(\cos\gamma-1)]}{(n-\zeta)(\cos\gamma-1)} \\ &{}+\sum_{n=2}^{\infty}\frac{\ln^{-1-\rho}[(n-\zeta)(\cos\gamma +1)]}{(n-\zeta)(\cos\gamma+1)} \\ =&\sum_{n=2}^{\infty} \biggl\{ \frac{\ln^{-1-\rho}[(n+\zeta)(1-\cos \gamma)]}{(n+\zeta)(1-\cos\gamma)}+\frac{\ln^{-1-\rho}[(n-\zeta )(\cos \gamma+1)]}{(n-\zeta)(\cos\gamma+1)} \biggr\} \\ \leq& \int_{\frac{3}{2}}^{\infty} \biggl\{ \frac{\ln^{-1-\rho }[(y+\zeta )(1-\cos\gamma)]}{(y+\zeta)(1-\cos\gamma)}+ \frac{\ln^{-1-\rho }[(y-\zeta)(\cos\gamma+1)]}{(y-\zeta)(\cos\gamma+1)} \biggr\} \,dy \\ =&\frac{1}{\rho} \biggl\{ \frac{\ln^{-\rho}[(\frac{3}{2}+\zeta )(1-\cos \gamma)]}{1-\cos\gamma}+\frac{\ln^{-\rho}[(\frac{3}{2}-\zeta )(1+\cos \gamma)]}{1+\cos\gamma} \biggr\} \\ =&\frac{1}{\rho} \biggl\{ \frac{1}{1-\cos\gamma}+\frac {1}{1+\cos\gamma}+o_{1}(1) \biggr\} \quad \bigl(\rho\rightarrow0^{+} \bigr). \end{aligned}$$

By (9), we still can find that

$$\begin{aligned} &h_{\rho}(\zeta,\gamma) \\ &\quad =\sum_{n=2}^{\infty} \biggl\{ \frac{\ln^{-1-\rho}[(n+\zeta)(1-\cos \gamma)]}{(n+\zeta)(1-\cos\gamma)}+\frac{\ln^{-1-\rho}[(n-\zeta )(\cos \gamma+1)]}{(n-\zeta)(\cos\gamma+1)} \biggr\} \\ &\quad \geq \int_{2}^{\infty} \biggl\{ \frac{\ln^{-1-\rho}[(y+\zeta)(1-\cos \gamma)]}{(y+\zeta)(1-\cos\gamma)}+ \frac{\ln^{-1-\rho}[(y-\zeta)(\cos \gamma+1)]}{(y-\zeta)(\cos\gamma+1)} \biggr\} \,dy \\ &\quad = \frac{1}{\rho} \biggl\{ \frac{\ln^{-\rho}[(2+\zeta)(1-\cos \gamma)]}{1-\cos\gamma}+\frac{\ln^{-\rho}[(2-\zeta)(1+\cos\gamma )]}{1+\cos \gamma} \biggr\} \\ &\quad = \frac{1}{\rho} \biggl\{ \frac{1}{1-\cos\gamma}+\frac {1}{1+\cos\gamma}+o_{2}(1) \biggr\} \quad \bigl(\rho\rightarrow0^{+} \bigr). \end{aligned}$$

Hence, we prove that (16) is valid. □

## Main results and a few particular cases

We also define

17 $$ H(\lambda_{1}):=H_{\beta}^{1/p}(\lambda _{1})H_{\alpha}^{1/q}(\lambda_{1})= \frac{2\pi\csc^{2/p}\beta\csc^{2/q}\alpha}{\lambda\sin(\frac{\pi\lambda_{1}}{\lambda})}. $$

### Theorem 1

*Suppose that* $a_{m},b_{n}\geq0$ ($\vert m \vert , \vert n \vert \in\mathbf{N}\backslash\{1\}$) *and*

$$\begin{aligned} &0 < \sum_{ \vert m \vert =2}^{\infty}\frac{\ln^{p(1-\lambda _{1})-1}A_{\alpha}(\xi,m)}{(A_{\alpha}(\xi,m))^{1-p}}a_{m}^{p}< \infty, \\ &0 < \sum_{ \vert n \vert =2}^{\infty}\frac{\ln^{q(1-\lambda _{2})-1}B_{\beta}(\eta,n)}{(B_{\beta}(\eta,n))^{1-q}}b_{n}^{q}< \infty. \end{aligned}$$

*We have the following reverse equivalent inequalities*:

18 $$\begin{aligned} &\begin{aligned}[b] I:={}&\sum_{ \vert n \vert =2}^{\infty} \sum_{ \vert m \vert =2}^{\infty}\frac{1}{\ln^{\lambda}A_{\alpha}(\xi,m)+\ln^{\lambda }B_{\beta}(\eta,n)}a_{m}b_{n} \\ >{}&H(\lambda_{1}) \Biggl[ \sum_{ \vert m \vert =2}^{\infty } \bigl(1-\theta(\lambda_{2},m) \bigr)\frac{\ln^{p(1-\lambda _{1})-1}A_{\alpha}(\xi ,m)}{(A_{\alpha}(\xi,m))^{1-p}}a_{m}^{p} \Biggr] ^{\frac{1}{p}} \\ &{}\times \Biggl[ \sum_{ \vert n \vert =2}^{\infty} \frac{\ln ^{q(1-\lambda_{2})-1}B_{\beta}(\eta,n)}{(B_{\beta}(\eta,n))^{1-q}}b_{n}^{q} \Biggr] ^{\frac{1}{q}}, \end{aligned} \end{aligned}$$

19 $$\begin{aligned} &\begin{aligned}[b] J :={}& \Biggl[ \sum_{ \vert n \vert =2}^{\infty} \frac{\ln^{p\lambda _{2}-1}B_{\beta}(\eta,n)}{B_{\beta}(\eta,n)} \Biggl( \sum_{ \vert m \vert =2}^{\infty} \frac{a_{m}}{\ln^{\lambda}A_{\alpha}(\xi ,m)+\ln^{\lambda}B_{\beta}(\eta,n)} \Biggr) ^{p} \Biggr] ^{\frac {1}{p}} \\ >{}&H(\lambda_{1}) \Biggl[ \sum_{ \vert m \vert =2}^{\infty } \bigl(1-\theta(\lambda_{2},m) \bigr)\frac{\ln^{p(1-\lambda _{1})-1}A_{\alpha}(\xi ,m)}{(A_{\alpha}(\xi,m))^{1-p}}a_{m}^{p} \Biggr] ^{1/p}, \end{aligned} \end{aligned}$$

20 $$\begin{aligned} &\begin{aligned}[b] L :={}& \Biggl[ \sum_{ \vert m \vert =2}^{\infty} \frac{\ln^{q\lambda _{1}-1}A_{\alpha}(\xi,m)}{(1-\theta(\lambda_{2},m))^{q-1}A_{\alpha }(\xi ,m)} \\ &{}\times \Biggl( \sum_{ \vert n \vert =2}^{\infty} \frac{1}{\ln ^{\lambda}A_{\alpha}(\xi,m)+\ln^{\lambda}B_{\beta}(\eta,n)}b_{n} \Biggr) ^{q} \Biggr] ^{\frac{1}{q}} \\ >{}&H(\lambda_{1}) \Biggl[ \sum_{ \vert n \vert =2}^{\infty} \frac{\ln^{q(1-\lambda_{2})-1}B_{\beta}(\eta,n)}{(B_{\beta}(\eta ,n))^{1-q}}b_{n}^{q} \Biggr] ^{1/q}. \end{aligned} \end{aligned}$$

*In particular*, (i) *for* $\alpha=\beta=\frac{\pi}{2}$, $\xi,\eta\in {}[ 0,\frac{1}{2}]$, *setting*

21 $$\begin{aligned} \theta_{1}(\lambda_{2},m) :=&\frac{\lambda}{\pi}\sin \biggl(\frac{\pi\lambda _{1}}{\lambda} \biggr) \int_{0}^{\frac{\ln(2+\eta)}{\ln \vert m-\xi \vert }}\frac{u^{\lambda_{2}-1}}{1+u^{\lambda}}\,du \\ =&O \biggl( \frac{1}{\ln^{\lambda_{2}} \vert m-\xi \vert } \biggr) \in(0,1), \end{aligned}$$

*we have the following reverse equivalent inequalities*:

22 $$\begin{aligned} &\begin{aligned}[b] &\sum_{ \vert n \vert =2}^{\infty}\sum _{ \vert m \vert =2}^{\infty}\frac{1}{\ln^{\lambda} \vert m-\xi \vert +\ln ^{\lambda} \vert n-\eta \vert }a_{m}b_{n} \\ &\quad >\frac{2\pi}{\lambda\sin(\frac{\pi\lambda_{1}}{\lambda})} \Biggl[ \sum_{ \vert m \vert =2}^{\infty} \bigl(1-\theta_{1}(\lambda_{2},m) \bigr)\frac{\ln^{p(1-\lambda_{1})-1} \vert m-\xi \vert }{ \vert m-\xi \vert ^{1-p}}a_{m}^{p} \Biggr] ^{\frac{1}{p}} \\ &\qquad {}\times \Biggl[ \sum_{ \vert n \vert =2}^{\infty} \frac{\ln ^{q(1-\lambda_{2})-1} \vert n-\eta \vert }{ \vert n-\eta \vert ^{1-q}}b_{n}^{q} \Biggr] ^{\frac{1}{q}}, \end{aligned} \end{aligned}$$

23 $$\begin{aligned} &\begin{aligned}[b] & \Biggl\{ \sum_{ \vert n \vert =2}^{\infty} \frac{\ln^{p\lambda _{2}-1} \vert n-\eta \vert }{ \vert n-\eta \vert } \Biggl[ \sum_{ \vert m \vert =2}^{\infty} \frac{a_{m}}{\ln^{\lambda } \vert m-\xi \vert +\ln^{\lambda} \vert n-\eta \vert } \Biggr] ^{p} \Biggr\} ^{\frac{1}{p}} \\ &\quad >\frac{2\pi}{\lambda\sin(\frac{\pi\lambda_{1}}{\lambda})} \Biggl[ \sum_{ \vert m \vert =2}^{\infty} \bigl(1-\theta_{1}(\lambda_{2},m) \bigr)\frac{\ln^{p(1-\lambda_{1})-1} \vert m-\xi \vert }{ \vert m-\xi \vert ^{1-p}}a_{m}^{p} \Biggr] ^{\frac{1}{p}}, \end{aligned} \end{aligned}$$

24 $$\begin{aligned} &\begin{aligned}[b] & \Biggl[ \sum_{ \vert m \vert =2}^{\infty} \frac{\ln^{q\lambda _{1}-1} \vert m-\xi \vert }{(1-\theta_{1}(\lambda_{2},m))^{q-1} \vert m-\xi \vert } \Biggl( \sum_{ \vert n \vert =2}^{\infty} \frac{1}{\ln ^{\lambda} \vert m-\xi \vert +\ln^{\lambda} \vert n-\eta \vert }b_{n} \Biggr) ^{q} \Biggr] ^{\frac{1}{q}} \\ &\quad >\frac{2\pi}{\lambda\sin(\frac{\pi\lambda_{1}}{\lambda})} \Biggl[ \sum_{ \vert n \vert =2}^{\infty} \frac{\ln^{q(1-\lambda _{2})-1} \vert n-\eta \vert }{ \vert n-\eta \vert ^{1-q}}b_{n}^{q} \Biggr] ^{\frac{1}{q}}. \end{aligned} \end{aligned}$$

(ii) *For* $\xi,\eta=0$, $\alpha,\beta\in{[} \arccos\frac{1}{3},\frac{\pi}{2}]$, *setting*

25 $$\begin{aligned} \theta_{2}(\lambda_{2},m) :=&\frac{\lambda}{\pi}\sin \biggl(\frac{\pi\lambda _{1}}{\lambda} \biggr) \int_{0}^{\frac{\ln2(1+\cos\beta)}{\ln( \vert m \vert +m\cos \alpha)}}\frac{u^{\lambda_{2}-1}}{1+u^{\lambda}}\,du \\ =&O \biggl( \frac{1}{\ln^{\lambda_{2}}( \vert m \vert +m\cos \alpha)} \biggr) \in(0,1), \end{aligned}$$

*we have the following equivalent inequalities*:

26 $$\begin{aligned} &\begin{aligned}[b] &\sum_{ \vert n \vert =2}^{\infty}\sum _{ \vert m \vert =2}^{\infty}\frac{a_{m}b_{n}}{\ln^{\lambda}( \vert m \vert +m\cos\alpha)+\ln^{\lambda}( \vert n \vert +n\cos\beta)} \\ &\quad >H(\lambda_{1}) \Biggl[ \sum_{ \vert m \vert =2}^{\infty } \bigl(1-\theta_{2}(\lambda_{2},m) \bigr) \frac{\ln^{p(1-\lambda_{1})-1}( \vert m \vert +m\cos\alpha)}{( \vert m \vert +m\cos\alpha)^{1-p}} a_{m}^{p} \Biggr] ^{1/p} \\ &\qquad {}\times \Biggl[ \sum_{ \vert n \vert =2}^{\infty} \frac{\ln ^{q(1-\lambda_{2})-1}( \vert n \vert +n\cos\beta)}{( \vert n \vert +n\cos\beta)^{1-q}}b_{n}^{q} \Biggr] ^{1/q}, \end{aligned} \end{aligned}$$

27 $$\begin{aligned} &\begin{aligned}[b] & \Biggl\{ \sum_{ \vert n \vert =2}^{\infty} \frac{\ln^{p\lambda _{2}-1}( \vert n \vert +n\cos\beta)}{ \vert n \vert +n\cos\beta} \Biggl[ \sum_{ \vert m \vert =2}^{\infty} \frac{a_{m}}{\ln^{\lambda}( \vert m \vert +m\cos\alpha)+\ln^{\lambda }( \vert n \vert +n\cos\beta)} \Biggr] ^{p} \Biggr\} ^{\frac {1}{p}} \\ &\quad >H(\lambda_{1}) \Biggl[ \sum_{ \vert m \vert =2}^{\infty } \bigl(1-\theta_{2}(\lambda_{2},m) \bigr) \frac{\ln^{p(1-\lambda_{1})-1}( \vert m \vert +m\cos\alpha)}{( \vert m \vert +m\cos\alpha)^{1-p}} a_{m}^{p} \Biggr] ^{\frac{1}{p}}, \end{aligned} \end{aligned}$$

28 $$\begin{aligned} &\begin{aligned}[b] & \Biggl[ \sum_{ \vert m \vert =2}^{\infty} \frac{\ln^{q\lambda _{1}-1}( \vert m \vert +m\cos\alpha)}{(1-\theta_{2}(\lambda _{2},m))^{q-1}( \vert m \vert +m\cos \alpha)} \\ &\qquad {}\times \Biggl( \sum_{ \vert n \vert =2}^{\infty} \frac{1}{\ln ^{\lambda}( \vert m \vert +m\cos\alpha)+\ln^{\lambda}( \vert n \vert +n\cos\beta)}b_{n} \Biggr) ^{q} \Biggr] ^{\frac{1}{q}} \\ &\quad > H(\lambda_{1}) \Biggl[ \sum_{ \vert n \vert =2}^{\infty} \frac{\ln^{q(1-\lambda _{2})-1}( \vert n \vert +n\cos\beta)}{( \vert n \vert +n\cos \beta)^{1-q}}b_{n}^{q} \Biggr] ^{\frac{1}{q}}. \end{aligned} \end{aligned}$$

### Proof

By the reverse Hölder inequality with weight (cf. [28]) and (8), we find

$$\begin{aligned} & \Biggl( \sum_{ \vert m \vert =2}^{\infty}H(m,n)a_{m} \Biggr) ^{p} \\ &\quad = \Biggl\{ \sum_{ \vert m \vert =2}^{\infty}H(m,n) \biggl[ \frac{(A_{\alpha}(\xi,m))^{1/q}\ln^{(1-\lambda_{1})/q}A_{\alpha}(\xi ,m)}{\ln ^{(1-\lambda_{2})/p}B_{\beta}(\eta,n)}a_{m} \biggr] \\ &\qquad {}\times \biggl[ \frac{\ln^{(1-\lambda_{2})/p}B_{\beta}(\eta,n)}{(A_{\alpha}(\xi,m))^{1/q}\ln^{(1-\lambda_{1})/q}A_{\alpha}(\xi,m)} \biggr] \Biggr\} ^{p} \\ &\quad \geq \sum_{ \vert m \vert =2}^{\infty}H(m,n) \frac{(A_{\alpha }(\xi,m))^{p/q}\ln^{(1-\lambda_{1})p/q}A_{\alpha}(\xi,m)}{\ln ^{1-\lambda_{2}}B_{\beta}(\eta,n)}a_{m}^{p} \\ &\qquad {}\times \Biggl[ \sum_{ \vert m \vert =2}^{\infty}H(m,n) \frac{\ln ^{(1-\lambda_{2})q/p}B_{\beta}(\eta,n)}{A_{\alpha}(\xi,m)\ln ^{1-\lambda_{1}}A_{\alpha}(\xi,m)} \Biggr] ^{p-1} \\ &\quad =\frac{(\varpi(\lambda_{1},n))^{p-1}B_{\beta}(\eta,n)}{\ln ^{p\lambda _{2}-1}B_{\beta}(\eta,n)}\sum_{ \vert m \vert =2}^{\infty} \frac{H(m,n)(A_{\alpha}(\xi,m))^{\frac{p}{q}}\ln^{(1-\lambda_{1})\frac {p}{q}}A_{\alpha}(\xi,m)}{B_{\beta}(\eta,n)\ln^{1-\lambda_{2}}B_{\beta }(\eta,n)}a_{m}^{p}. \end{aligned}$$

By (14), in view of $p-1<0$, it follows that

29 $$\begin{aligned} J >&H_{\alpha}^{1/q}(\lambda_{1}) \Biggl[ \sum _{ \vert n \vert =2}^{\infty}\sum_{ \vert m \vert =2}^{\infty} \frac{H(m,n)(A_{\alpha}(\xi,m))^{p/q}\ln^{(1-\lambda_{1})p/q}A_{\alpha }(\xi ,m)}{B_{\beta}(\eta,n)\ln^{1-\lambda_{2}}B_{\beta}(\eta ,n)}a_{m}^{p} \Biggr] ^{\frac{1}{p}} \\ =&H_{\alpha}^{1/q}(\lambda_{1}) \Biggl[ \sum _{ \vert m \vert =2}^{\infty}\sum_{ \vert n \vert =2}^{\infty} \frac{H(m,n)(A_{\alpha}(\xi,m))^{p/q}\ln^{(1-\lambda_{1})p/q}A_{\alpha }(\xi ,m)}{B_{\beta}(\eta,n)\ln^{1-\lambda_{2}}B_{\beta}(\eta ,n)}a_{m}^{p} \Biggr] ^{\frac{1}{p}} \\ =&H_{\alpha}^{1/q}(\lambda_{1}) \Biggl[ \sum _{ \vert m \vert =2}^{\infty}\omega(\lambda_{2},m) \frac{\ln^{p(1-\lambda _{1})-1}A_{\alpha}(\xi,m)}{(A_{\alpha}(\xi,m))^{1-p}}a_{m}^{p} \Biggr] ^{\frac{1}{p}}. \end{aligned}$$

By (11) and (17), we have (19).

Using the reverse Hölder inequality again, we have

30 $$\begin{aligned} I =&\sum_{ \vert n \vert =2}^{\infty} \Biggl[ \frac{\ln^{\lambda _{2}-(1/p)}B_{\beta}(\eta,n)}{(B_{\beta}(\eta,n))^{1/p}}\sum_{ \vert m \vert =2}^{\infty}H(m,n)a_{m} \Biggr] \biggl[ \frac{\ln ^{(1/p)-\lambda_{2}}B_{\beta}(\eta,n)}{(B_{\beta}(\eta ,n))^{-1/p}}b_{n} \biggr] \\ \geq&J \Biggl[ \sum_{ \vert n \vert =2}^{\infty} \frac{\ln ^{q(1-\lambda_{2})-1}B_{\beta}(\eta,n)}{(B_{\beta}(\eta,n))^{1-q}}b_{n}^{q} \Biggr] ^{\frac{1}{q}}, \end{aligned}$$

and then by (19) we have (18).

On the other hand, assuming that (18) is valid, we set

$$ b_{n}:=\frac{\ln^{p\lambda_{2}-1}B_{\beta}(\eta,n)}{B_{\beta }(\eta,n)} \Biggl( \sum _{ \vert m \vert =2}^{\infty}H(m,n)a_{m} \Biggr) ^{p-1},\quad \vert n \vert \in\mathbf{N}\backslash\{1\}, $$

and find

$$ J= \Biggl[ \sum_{ \vert n \vert =2}^{\infty} \frac{\ln^{q(1-\lambda _{2})-1}B_{\beta}(\eta,n)}{(B_{\beta}(\eta,n))^{1-q}}b_{n}^{q} \Biggr] ^{\frac{1}{p}}. $$

By (29) it follows that $J>0$. If $J=\infty$, then (19) is trivially valid; if $J<\infty$, then we have

$$\begin{aligned} &\begin{aligned} &\sum_{ \vert n \vert =2}^{\infty} \frac{\ln^{q(1-\lambda _{2})-1}B_{\beta}(\eta,n)}{(B_{\beta}(\eta,n))^{1-q}}b_{n}^{q} \\ &\quad =J^{p}=I \\ &\quad >H(\lambda_{1}) \Biggl[ \sum_{ \vert m \vert =2}^{\infty } \bigl(1-\theta(\lambda_{2},m) \bigr)\frac{\ln^{p(1-\lambda _{1})-1}A_{\alpha}(\xi ,m)}{(A_{\alpha}(\xi,m))^{1-p}}a_{m}^{p} \Biggr] ^{\frac{1}{p}} \\ &\qquad {}\times \Biggl[ \sum_{ \vert n \vert =2}^{\infty} \frac{\ln ^{q(1-\lambda_{2})-1}B_{\beta}(\eta,n)}{(B_{\beta}(\eta,n))^{1-q}}b_{n}^{q} \Biggr] ^{\frac{1}{q}}, \end{aligned} \\ &\begin{aligned} J &= \Biggl[ \sum_{ \vert n \vert =2}^{\infty} \frac{\ln ^{q(1-\lambda_{2})-1}B_{\beta}(\eta,n)}{(B_{\beta}(\eta,n))^{1-q}}b_{n}^{q} \Biggr] ^{\frac{1}{p}} \\ &> H(\lambda_{1}) \Biggl[ \sum_{ \vert m \vert =2}^{\infty } \bigl(1-\theta(\lambda_{2},m) \bigr)\frac{\ln^{p(1-\lambda _{1})-1}A_{\alpha}(\xi ,m)}{(A_{\alpha}(\xi,m))^{1-p}}a_{m}^{p} \Biggr] ^{\frac{1}{p}}. \end{aligned} \end{aligned}$$

Hence (19) is valid, which is equivalent to (18).

We have proved that (18) is valid. Then we set

$$ a_{m}:= \frac{\ln^{q\lambda_{1}-1}A_{\alpha}(\xi,m)}{(1-\theta (\lambda _{2},m))^{q-1}A_{\alpha}(\xi,m)} \Biggl( \sum _{ \vert n \vert =2}^{\infty}H(m,n)b_{n} \Biggr) ^{q-1},\quad \vert m \vert \in\mathbf{N}\backslash\{ 1\}, $$

and find

$$ L= \Biggl[ \sum_{ \vert m \vert =2}^{\infty} \bigl(1-\theta( \lambda_{2},m) \bigr)\frac{\ln^{p(1-\lambda_{1})-1}A_{\alpha}(\xi ,m)}{(A_{\alpha }(\xi,m))^{1-p}}a_{m}^{p} \Biggr] ^{\frac{1}{q}}. $$

If $L=0$, then (20) is impossible, namely $L>0$. If $L=\infty $, then (20) is trivially valid; if $L<\infty$, then we have

$$\begin{aligned} &\begin{aligned} &\sum_{ \vert m \vert =2}^{\infty} \bigl(1- \theta(\lambda _{2},m) \bigr)\frac{\ln^{p(1-\lambda_{1})-1}A_{\alpha}(\xi,m)}{(A_{\alpha}(\xi ,m))^{1-p}}a_{m}^{p} \\ &\quad =L^{q}=I \\ &\quad >H(\lambda_{1}) \Biggl[ \sum_{ \vert m \vert =2}^{\infty } \bigl(1-\theta(\lambda_{2},m) \bigr)\frac{\ln^{p(1-\lambda _{1})-1}A_{\alpha}(\xi ,m)}{(A_{\alpha}(\xi,m))^{1-p}}a_{m}^{p} \Biggr] ^{\frac{1}{p}} \\ &\qquad {}\times \Biggl[ \sum_{ \vert n \vert =2}^{\infty} \frac{\ln ^{q(1-\lambda_{2})-1}B_{\beta}(\eta,n)}{(B_{\beta}(\eta,n))^{1-q}}b_{n}^{q} \Biggr] ^{\frac{1}{q}}, \end{aligned} \\ &\begin{aligned} L &= \Biggl[ \sum_{ \vert m \vert =2}^{\infty} \bigl(1-\theta (\lambda_{2},m) \bigr)\frac{\ln^{p(1-\lambda_{1})-1}A_{\alpha}(\xi ,m)}{(A_{\alpha }(\xi,m))^{1-p}}a_{m}^{p} \Biggr] ^{\frac{1}{q}} \\ &>H(\lambda_{1}) \Biggl[ \sum_{ \vert n \vert =2}^{\infty} \frac{\ln^{q(1-\lambda_{2})-1}B_{\beta}(\eta,n)}{(B_{\beta}(\eta ,n))^{1-q}}b_{n}^{q} \Biggr] ^{\frac{1}{q}}. \end{aligned} \end{aligned}$$

Hence, (20) is valid.

On the other hand, assuming that (20) is valid, using the reverse Hölder inequality, we have

31 $$\begin{aligned} I =&\sum_{ \vert m \vert =2}^{\infty} \biggl[ \frac{\ln ^{(1/q)-\lambda_{1}}A_{\alpha}(\xi,m)}{(1-\theta(\lambda _{2},m))^{-1/p}(A_{\alpha}(\xi,m))^{-1/q}}a_{m} \biggr] \\ &{}\times \Biggl[ \frac{\ln^{\lambda_{1}-(1/q)}A_{\alpha}(\xi,m)}{(1-\theta(\lambda_{2},m))^{1/p}(A_{\alpha}(\xi,m))^{1/q}}\sum _{ \vert n \vert =2}^{\infty}H(m,n)b_{n} \Biggr] \\ \geq& \Biggl[ \sum_{ \vert m \vert =2}^{\infty} \bigl(1- \theta(\lambda_{2},m) \bigr)\frac{\ln^{p(1-\lambda _{1})-1}A_{\alpha}(\xi,m)}{(A_{\alpha }(\xi,m))^{1-p}}a_{m}^{p} \Biggr] ^{\frac{1}{p}}L, \end{aligned}$$

and then by (20) we have (18), which is equivalent to (20).

Therefore, inequalities (18), (19), and (20) are equivalent.

The theorem is proved. □

### Theorem 2

*With regards to the assumptions of Theorem *1, *the constant factor* $H(\lambda_{1})$ *in* (18), (19), *and* (20) *is the best possible*.

### Proof

For $0<\varepsilon<p\lambda_{1}$, we set $\widetilde {\lambda }_{1}=\lambda_{1}-\frac{\varepsilon}{p}$ ($\in(0,1)$), $\widetilde {\lambda}_{2}=\lambda_{2}+\frac{\varepsilon}{p}$ (>0), and

$$\begin{aligned} &\widetilde{a}_{m}:=\frac{\ln^{\lambda_{1}-(\varepsilon /p)-1}A_{\alpha }(\xi,m)}{A_{\alpha}(\xi,m)}=\frac{\ln^{\widetilde{\lambda}_{1}-1}A_{\alpha}(\xi,m)}{A_{\alpha}(\xi,m)}\quad \bigl( \vert m \vert \in\mathbf{N}\backslash\{1\} \bigr), \\ &\widetilde{b}_{n}:=\frac{\ln^{\lambda_{2}-(\varepsilon /q)-1}B_{\beta }(\eta,n)}{B_{\beta}(\eta,n)}=\frac{\ln^{\widetilde{\lambda}_{2}-\varepsilon-1}B_{\beta}(\eta,n)}{B_{\beta}(\eta,n)}\quad \bigl( \vert n \vert \in\mathbf{N}\backslash\{ 1\} \bigr). \end{aligned}$$

By (16) and (14) we find

$$\begin{aligned} &\begin{aligned} \widetilde{I}_{1} := {}& \Biggl[ \sum _{ \vert m \vert =2}^{\infty } \bigl(1-\theta(\lambda_{2},m) \bigr)\frac{\ln^{p(1-\lambda _{1})-1}A_{\alpha}(\xi ,m)}{(A_{\alpha}(\xi,m))^{1-p}} \widetilde{a}_{m}^{p} \Biggr] ^{\frac{1}{p}} \\ &{}\times \Biggl[ \sum_{ \vert n \vert =2}^{\infty} \frac{\ln ^{q(1-\lambda_{2})-1}B_{\beta}(\eta,n)}{(B_{\beta}(\eta,n))^{1-q}}\widetilde{b}_{n}^{q} \Biggr] ^{\frac{1}{q}} \\ ={}& \Biggl[ \sum_{ \vert m \vert =2}^{\infty} \frac{\ln ^{-1-\varepsilon}A_{\alpha}(\xi,m)}{A_{\alpha}(\xi,m)}-\sum_{ \vert m \vert =2}^{\infty} \frac{O(\ln^{-1-(\lambda_{2}+\varepsilon )}A_{\alpha}(\xi,m))}{A_{\alpha}(\xi,m)} \Biggr] ^{\frac{1}{p}} \\ &{}\times \Biggl[ \sum_{ \vert n \vert =2}^{\infty} \frac{\ln ^{-1-\varepsilon}B_{\beta}(\eta,n)}{B_{\beta}(\eta,n)} \Biggr] ^{\frac{1}{q}} \\ ={}&\frac{1}{\varepsilon} \bigl(2\csc^{2}\alpha+o(1)-\varepsilon O(1) \bigr)^{1/p} \bigl(2\csc^{2}\beta+\widetilde{o}(1) \bigr)^{1/q}\quad \bigl(\varepsilon\rightarrow0^{+} \bigr), \end{aligned} \\ &\begin{aligned} \widetilde{I} &:=\sum_{ \vert m \vert =2}^{\infty } \sum_{ \vert n \vert =2}^{\infty}H(m,n)\widetilde{a}_{m}\widetilde{b}_{n} \\ &=\sum_{ \vert n \vert =2}^{\infty}\sum _{ \vert m \vert =2}^{\infty}H(m,n)\frac{\ln^{\widetilde{\lambda}_{1}-1}A_{\alpha }(\xi ,m)\ln^{\widetilde{\lambda}_{2}-\varepsilon-1}B_{\beta}(\eta,n)}{A_{\alpha}(\xi,m)B_{\beta}(\eta,n)} \\ &=\sum_{ \vert n \vert =2}^{\infty}\varpi(\widetilde{ \lambda}_{1},n)\frac{\ln^{-1-\varepsilon}B_{\beta}(\eta,n)}{B_{\beta }(\eta,n)}< H_{\alpha}( \widetilde{\lambda}_{1})\sum_{ \vert n \vert =2}^{\infty} \frac{\ln^{-1-\varepsilon}B_{\beta}(\eta,n)}{B_{\beta }(\eta,n)} \\ &=\frac{1}{\varepsilon}H_{\alpha} \biggl(\lambda_{1}- \frac{\varepsilon}{p} \biggr) \bigl(2\csc^{2}\beta+ \widetilde{o}(1) \bigr). \end{aligned} \end{aligned}$$

If there exists a positive number $K\geq H(\lambda_{1})$ such that (18) is still valid when replacing $H(\lambda_{1})$ by *K*, then, in particular, we have

$$ \varepsilon\widetilde{I}=\varepsilon\sum_{ \vert m \vert =2}^{\infty} \sum_{ \vert n \vert =2}^{\infty}H(m,n)\widetilde{a}_{m} \widetilde{b}_{n}>\varepsilon K\widetilde{I}_{1}. $$

In view of the above results, it follows that

$$\begin{aligned} &H_{\alpha} \biggl(\lambda_{1}-\frac{\varepsilon}{p} \biggr) \bigl(2\csc^{2}\beta+\widetilde{o}(1) \bigr) >K\cdot \bigl(2 \csc^{2}\alpha+o(1)-\varepsilon O(1) \bigr)^{1/p} \bigl(2 \csc^{2}\beta+\widetilde{o}(1) \bigr)^{1/q}, \end{aligned}$$

and then

$$ \frac{4\pi\csc^{2}\alpha}{\lambda\sin(\frac{\pi\lambda _{1}}{\lambda})}\csc^{2}\beta\geq2K\csc^{2/p}\alpha \csc^{2/q}\beta \quad \bigl(\varepsilon\rightarrow0^{+} \bigr), $$

namely

$$ H(\lambda_{1})=\frac{2\pi}{\lambda\sin(\frac{\pi\lambda _{1}}{\lambda})}\csc^{2/p}\beta\csc ^{2/q}\alpha\geq K. $$

Hence, $K=H(\lambda_{1})$ is the best possible constant factor in (18).

The constant factor $H(\lambda_{1})$ in (19) ((20)) is still the best possible. Otherwise we would reach a contradiction by (30) ((31)) that the constant factor in (18) is not the best possible. □

### Remark 2

(i) For $\xi=\eta=0$ in (22), setting

$$\begin{aligned} \widetilde{\theta}_{1}(\lambda_{2},m) :=& \frac{\lambda}{\pi}\sin \biggl(\frac{\pi\lambda_{1}}{\lambda} \biggr) \int_{0}^{\frac{\ln2}{\ln \vert m \vert }}\frac{u^{\lambda _{2}-1}}{1+u^{\lambda}}\,du \\ =&O \biggl( \frac{1}{\ln^{\lambda_{2}} \vert m \vert } \biggr) \in(0,1), \end{aligned}$$

we have the following new inequality with the best possible constant factor $\frac{2\pi}{\lambda\sin(\frac{\pi\lambda_{1}}{\lambda})}$:

32 $$\begin{aligned} &\sum_{ \vert n \vert =2}^{\infty}\sum _{ \vert m \vert =2}^{\infty}\frac{a_{m}b_{n}}{\ln^{\lambda} \vert m \vert +\ln ^{\lambda} \vert n \vert } \\ &\quad >\frac{2\pi}{\lambda\sin(\frac{\pi\lambda_{1}}{\lambda})} \Biggl[ \sum_{ \vert m \vert =2}^{\infty} \bigl(1-\widetilde{\theta}_{1}(\lambda_{2},m) \bigr)\frac{\ln^{p(1-\lambda_{1})-1} \vert m \vert }{ \vert m \vert ^{1-p}}a_{m}^{p} \Biggr] ^{\frac{1}{p}} \\ &\qquad {}\times \Biggl[ \sum_{ \vert n \vert =2}^{\infty} \frac{\ln ^{q(1-\lambda_{2})-1} \vert n \vert }{ \vert n \vert ^{1-q}}b_{n}^{q} \Biggr] ^{\frac{1}{q}}. \end{aligned}$$

It follows that (20) ((22)) is an extension of (29).

(ii) If $a_{-m}=a_{m}$ and $b_{-n}=b_{n}$ ($m,n\in\mathbf{N}\backslash\{1\}$), setting

$$\begin{aligned} & \theta_{2}(\lambda_{2},m) :=\frac{\lambda}{\pi}\sin \biggl(\frac{\pi\lambda _{1}}{\lambda} \biggr) \int_{0}^{\frac{\ln(2+\eta)}{\ln(m-\xi)}}\frac{u^{\lambda_{2}-1}}{1+u^{\lambda}}\,du =O \biggl( \frac{1}{\ln^{\lambda_{2}}(m-\xi)} \biggr) \in(0,1), \\ & \theta_{3}(\lambda_{2},m) :=\frac{\lambda}{\pi}\sin \biggl(\frac{\pi\lambda _{1}}{\lambda} \biggr) \int_{0}^{\frac{\ln(2+\eta)}{\ln(m+\xi)}}\frac{u^{\lambda_{2}-1}}{1+u^{\lambda}}\,du =O \biggl( \frac{1}{\ln^{\lambda_{2}}(m+\xi)} \biggr) \in(0,1), \end{aligned}$$

then (22) reduces to

33 $$\begin{aligned} &\sum_{n=2}^{\infty}\sum _{m=2}^{\infty} \biggl[ \frac{1}{\ln^{\lambda }(m-\xi)+\ln^{\lambda}(n-\eta)}+ \frac{1}{\ln^{\lambda}(m-\xi)+\ln ^{\lambda}(n+\eta)} \\ &\qquad {} +\frac{1}{\ln^{\lambda}(m+\xi)+\ln^{\lambda}(n-\eta)}+\frac{1 }{\ln^{\lambda}(m+\xi)+\ln^{\lambda}(n+\eta)} \biggr] a_{m}b_{n} \\ &\quad >\frac{2\pi}{\lambda\sin(\pi\lambda_{1}/\lambda)} \Biggl\{ \sum_{m=2}^{\infty} \biggl[ \bigl(1-\theta_{2}(\lambda_{2},m) \bigr) \frac{\ln ^{p(1-\lambda_{1})-1}(m-\xi)}{(m-\xi)^{1-p}} \\ &\qquad {} + \bigl(1-\theta_{3}(\lambda_{2},m) \bigr) \frac{\ln^{p(1-\lambda _{1})-1}(m+\xi)}{(m+\xi)^{1-p}} \biggr] a_{m}^{p} \Biggr\} ^{\frac{1}{p}} \\ &\qquad {}\times \Biggl\{ \sum_{n=2}^{\infty} \biggl[ \frac{\ln^{q(1-\lambda _{2})-1}(n-\eta)}{(n-\eta)^{1-q}}+\frac{\ln^{q(1-\lambda _{2})-1}(n+\eta)}{(n+\eta)^{1-q}} \biggr] b_{n}^{q} \Biggr\} ^{\frac{1}{q}}. \end{aligned}$$

In particular, for $\xi=\eta=0$, $\lambda=1$, $\lambda_{1}=\lambda _{2}=\frac{1}{2}$ in (33), setting

$$ \theta(m):=\frac{1}{\pi} \int_{0}^{\frac{\ln2}{\ln m}}\frac{u^{-1/2}}{1+u}\,du=O \biggl( \frac{1}{\ln^{1/2}m} \biggr) \in(0,1), $$

we have the following reverse Mulholland inequality (2) with the best possible constant *π*:

34 $$\begin{aligned} \sum_{n=2}^{\infty}\sum _{m=2}^{\infty}\frac{a_{m}b_{n}}{\ln mn} >&\pi \Biggl[ \sum_{m=2}^{\infty} \bigl(1-\theta(m) \bigr) \frac{\ln^{\frac{p}{2}-1}m}{m^{1-p}}a_{m}^{p} \Biggr] ^{\frac{1}{p}} \\ &{} \times \Biggl( \sum_{n=2}^{\infty} \frac{\ln^{\frac{q}{2}-1}n}{n^{1-q}}b_{n}^{q} \Biggr) ^{\frac{1}{q}}. \end{aligned}$$

## Conclusions

In this paper, by introducing multi-parameters, applying the weight coefficients, and using Hermite–Hadamard’s inequality, we give a reverse of the extension of Mulholland’s inequality in the whole plane with the best possible constant factor in Theorems 1–2. The equivalent forms and a few particular cases are considered. The technique of real analysis is very important as it is the key to proving the reverse equivalent inequalities with the best possible constant factor. The lemmas and theorems provide an extensive account of this type of inequalities.
